# Bilberry (*Vaccinium myrtillus* L.) Powder Has Anticarcinogenic Effects on Oral Carcinoma In Vitro and In Vivo

**DOI:** 10.3390/antiox10081319

**Published:** 2021-08-22

**Authors:** Matti Mauramo, Tuulia Onali, Wafa Wahbi, Jenni Vasara, Anniina Lampinen, Elina Mauramo, Anne Kivimäki, Stefan Martens, Hely Häggman, Meeri Sutinen, Tuula Salo

**Affiliations:** 1 Department of Oral and Maxillofacial Diseases, University of Helsinki and Helsinki University Hospital, 00014 Helsinki, Finland; tuulia.onali@helsinki.fi (T.O.); wafa.wahbi@helsinki.fi (W.W.); jenni.vasara@gmail.com (J.V.); anne.kivimaki@helsinki.fi (A.K.); tuula.salo@helsinki.fi (T.S.); 2 Department of Pathology, Haartman Institute and HUSLAB, Helsinki University Central Hospital, 00260 Helsinki, Finland; 3 Medical Nutrition Physiology, Department of Pharmacology, Faculty of Medicine, University of Helsinki, 00100 Helsinki, Finland; 4 Department of Ecology and Genetics, University of Oulu, 90570 Oulu, Finland; anniina.lampinen@gmail.com (A.L.); hely.haggman@oulu.fi (H.H.); 5 Department of Public Health, University of Helsinki, 00014 Helsinki, Finland; elina.mauramo@helsinki.fi; 6 Fondazione Edmund Mach, Research and Innovation Center, TN, 380105 San Michele’all Adige, Italy; stefan.martens@fmach.it; 7 Cancer and Translational Medicine Research Unit, Medical Research Center Oulu, University of Oulu, 90570 Oulu, Finland; meeri.sutinen@gmail.com

**Keywords:** bilberry, oral cancer, carcinoma, anthocyanin, viability, proliferation, invasion, migration, metastasis, zebrafish, tumorigenesis

## Abstract

Previous studies indicate that bilberry with high amounts of phenolic compounds can inhibit carcinogenic processes of colorectal cancer in vitro and in vivo. However, no studies have focused on the effects of bilberry on oral cancer. In this study, we aimed to examine the effects of bilberry powder on oral squamous cell carcinoma (OSCC) cells using both in vitro and in vivo assays. The effects of 0, 1, 10, and 25 mg/mL of whole bilberry powder on the viability, proliferation, migration, and invasion of OSCC (HSC-3) cells were examined and compared with 0.01 mg/mL of cetuximab. Two oral keratinocyte cell lines served as controls. Tumor area was analyzed in zebrafish microinjected with HSC-3 cells and treated with 2.5, 10, or 25 µg/mL of bilberry powder. Metastases in the head or tail areas were counted. Bilberry powder inhibited the viability, proliferation, migration, and invasion of HSC-3 cells (*p* < 0.05), which was more pronounced with higher concentrations. Cetuximab had no effect on HSC-3 cell migration or invasion. Compared to controls, the tumor area in zebrafish treated with bilberry powder (10 and 25 µg/mL) was reduced significantly (*p* = 0.038 and *p* = 0.021, respectively), but the number of fish with metastases did not differ between groups. Based on our in vitro and in vivo experiments, we conclude that whole bilberry powder has anti-tumor effects on OSCC cells.

## 1. Introduction

Oral cancers are often in advanced stage when diagnosed and have poor prognosis [1]. The risk of developing cancer in the oral cavity is related to lifestyle and health factors, such as alcohol, tobacco and betel quid consumption, and HPV infection [1]. Oral epithelia are regularly exposed to dietary compounds, some of which could provide protection against cancer initiation and progression. Diets rich in plant phytochemicals are associated with a smaller risk of developing cancer, especially digestive tract cancers [2,3]. Wild berries contain high amounts of phytochemicals, such as polyphenols, and a vast amount of evidence indicates that berries have anticancer potential [4,5,6,7,8,9,10]. Berries of the genus *Vaccinium* (e.g., cranberry, blueberry, bilberry, and lingonberry) have been shown to inhibit carcinogenic processes in various types of carcinoma cells, including breast, ovarian, colon, and hepatocellular carcinomas [11,12,13,14,15]. As wild berries and especially bilberries are readily available and a part of traditional diets in Finland and other Northern countries [5,9], they can contribute to the dietary intake of phenolic compounds.

We recently systematically reviewed the anticancer effects of bilberry (*Vaccinium myrtillus* L.) on digestive tract cancers [16] and found that bilberry inhibits proliferation of colon cancer cells in vitro and formation and growth of tumors and pre-malignant lesions in murine models of colorectal cancer. However, no studies focusing on the effects of bilberry on oral cancers were found. In one recent study, cultivated blueberry inhibited oral carcinoma invasion and angiogenesis in a hamster model by blocking oncogenic signaling pathways [17]. Fermented juice of lingonberry, a close relative of bilberry, reduced the proliferation and invasion of oral cancer cells in vitro [18]. Additionally, grape seed- and black raspberry-derived powders, high in proanthocyanidins, one of the main phenolic compounds in bilberry, have shown anticarcinogenic potential against oral cancer cells [19,20,21,22]. One clinical trial showed that mucoadhesive black raspberry gel can provide effective protection against premalignant oral intraepithelial lesions in terms of a statistically significant reduction in lesion size, histologic grade, and loss of heterozygosity [22].

The health benefits of *Vaccinium* berries are attributed to phenolic compounds synthesized via the phenylpropanoid pathway [23,24]. Wild-growing bilberry is rich in phenolic compounds, including flavonoids, such as flavonols, proanthocyanidins, and especially anthocyanins [23,24,25,26,27,28]. In addition to anticancer effects, anthocyanins have anti-inflammatory, antimutagenic, antimicrobial, antiobesity, and antioxidant properties [24,29,30]. Several studies have emphasized the role of growth conditions in the northern latitudes, leading to higher levels of phenolic compounds, especially anthocyanidins (hydrophobic aglycons of bilberry anthocyanins) compared to more southern habitats [31,32].

In this study, the effects of whole bilberry powder on OSCC cells were examined *in vitro*, and with a fast and low-cost zebrafish model in vivo [33]. The observed in vitro effects on invasive cells were compared with the effects of bilberry powder on keratinocyte cell lines. In addition, the effectivity of bilberry powder on the invasive cell line was compared with cetuximab. Cetuximab is a commercially available monoclonal antibody of epidermal growth factor, capable of promoting apoptosis and inhibiting the cell cycle, and approved by the FDA in March 2006 for treating squamous cell carcinoma of the head and neck [34]. The anthocyanin content of the whole bilberry powder was also examined. The hypothesis of this study was that bilberry powder inhibits the viability, proliferation, and invasion of OSCC cells in vitro and tumorigenesis and metastasis *in vivo*. Cetuximab was expected to be more potent (25). Our experiments showed that whole bilberry powder had antitumor effects in vitro and in vivo. Bilberry was even more effective than cetuximab on aggressive HSC-3 cells in vitro with the concentrations used in this study. In addition, HSC-3 were more sensitive to the inhibitory effects of bilberry than oral keratinocytes.

## 2. Materials and Methods

### 2.1. Cells and Cell Cultures

The invasive oral carcinoma cells used in the assays were highly malignant HSC-3 tongue squamous cell carcinoma cells (Japan Health Science Resources Bank, Osaka, Japan, JRCB 0623). The non-malignant epithelial cells were (1) spontaneously immortalized human oral mucosal keratinocytes (HMKs) obtained from surgical gingival biopsy and (2) human papilloma virus transformed oral epithelial cells (IHGKs) [35,36]. The cell lines were cultured in their normal growth conditions as described earlier [35,36,37].

### 2.2. Bilberry Powder

Whole bilberry (*Vaccinium myrtillus* L.) powder was provided by the berry company Kiantama, Finland (http://www.kiantama.fi/company/; accessed on 5 July 2021). Bilberries were picked in the south-east part of Finland (Juva and Luumäki regions) and stored frozen (<−18 °C), transferred to the drier shelves (42–45 °C) of the drying facility for 1 day, followed by homogenization into a powder, sieving through 1.5 mm/mesh, and packaging. During the process, the moisture content was monitored and microbiological analyses were conducted before release and delivery. The product and lot numbers for the whole berry powder were 170,100 and 1,636,300, respectively.

In the laboratory, bilberry powder was dissolved to phosphate-buffered saline (PBS) at 100 mg/mL concentration. During dissolving, the bilberry powder–PBS mixture was briefly vortexed and thereafter mixed in a shaker. After centrifugation, the solution was suction filtrated, syringe filtrated with a 0.45-µm filter, and finally sterile filtrated with a 0.22-µm filter in a laminar flow hood. The obtained bilberry solution was stored in 1-mL aliquots at −70 °C until further use (assays and analytics) to avoid degradation of anthocyanins. The concentrations used in the in vitro studies (0.2, 1, 5, 10, and 25 mg/mL) were based on previous studies on berry preparations and digestive system cancer cell lines [38,39,40,41] as well as on our own preliminary viability and proliferation assays (data not shown). The concentrations used in the zebrafish experiment (2.5, 10, or 25 µg/mL) were based on our previous pilot study (data not shown).

### 2.3. Anthocyanin Analysis

The analysis of anthocyanins of the bilberry powder stock solution was performed according to a previous study [42]. The method has also been applied successfully for bilberry [43]. Chromatography, quantification, and mass spectrometry conditions can be found in the literature referred above [42].

### 2.4. Epidermal Growth Factor Receptor Inhibitor

As a comparison to bilberry, a commercially available epidermal growth factor receptor (EGFR) inhibitor, cetuximab (Erbitux^®^), was used at a concentration of 0.01 mg/mL based on previous experiments with different concentrations of cetuximab [44].

### 2.5. Preparation of Myogel

Myogel coating was used in the IncuCyte migration and invasion assays. The human uterus leiomyoma tumor-derived matrix Myogel was prepared as previously described, similarly to Engelbreth–Holm–Swarm (EHS) mouse sarcoma-derived commercial products, such as Matrigel^®^ [45]. Myoma tissue was ground to a powder, suspended in a sodium chloride buffer, and after centrifugation, the pellet was homogenized in the same buffer followed by protein concentration measurement.

### 2.6. Cell Viability Assay

Cell viability was determined with the commercial, MTT-based, Cell Growth Determination Kit, (Sigma-Aldrich, St Louis, MO, USA) according to the product protocol. Briefly, cells were cultured in 96-well plates at a density of 5 × 10^3^ cells in the normal culture medium (100 μL) for 24 h after which the medium (75 μL) was changed and the cells were exposed to 0.2, 1, 5, 10, and 25 mg/mL of bilberry added in 25 µL of PBS. Phosphate-buffered saline (PBS 25 µL/100 µL) was used as a control. After 24 or 72 h of incubation, MTT solution was added and incubation continued for 3–4 h. Culture media was removed, and the cell cultures washed twice with PBS before adding isopropanol (MTT solvent) to avoid the effects of anthocyanin pigments on the measurements with an ELISA reader at 550 nm. There were two (HSC-3 cells) or three (HMK and IHGK cells) independent experiments with six replicates in each assay.

### 2.7. Cell Proliferation Assay

Cell proliferation was measured with the commercial Cell Proliferation ELISA, BrdU (colorimetric) kit (Roche Diagnostics, Basel, Switzerland) according to the manufacturer’s instructions. Briefly, cells were cultured in 96-well plates at a density of 5 × 10^3^ cells in normal culture medium (100 μL) for 24 h, after which the culture medium was changed and the cells exposed to 0.2, 1, 5, 10, and 25 mg/mL of bilberry. PBS (25 µL/100 µL) was used as a control. After 24- and 72-h incubations, the cells were labelled using 10 μM BrdU per well and re-incubated for 2 h at 37 °C in a humidified atmosphere. Culture medium was removed, cells were fixed, and DNA was denatured in one step by adding FixDenat (Roche Diagnostics, Basel, Switzerland). Next, the cells were incubated with anti-BrdU-POD for 90 min at room temperature. After removal of the antibody conjugate, cells were washed twice, and substrate solution was added. The reaction product was quantified by measuring absorbance using a scanning multi-well spectrophotometer (Thermo Scientific Multiskan EX, Thermo Fisher Scientific, Waltham, MA, USA) at 450 nm with a reference wavelength of 690 nm. There were two (HSC-3 cells) or three (HMK and IHGK cells) independent experiments with six replicates in each assay.

### 2.8. Transmigration Assay

In total, 500 μL of complete medium were first added to the lower chamber of a Transwell^®^ insert (Corning Inc., Corning, NY, USA). Then, 50,000 HSC-3 cells were suspended in 100 μL of medium with 0.5% lactalbumin instead of serum and seeded onto the Transwell^®^ nylon filter membrane insert. Study reagents or PBS as a control were added to the media in both the upper and lower chambers. Transwell^®^ inserts were incubated for 72 h at 37 °C in a 5% CO_2_ humidified atmosphere, after which the cells were fixed in 4% formaldehyde for 1 h at room temperature. Inserts were then washed with PBS. The cells were stained by adding 500 µL of 1% Toluidine blue in 1% Borax to the wells and incubated for 10 min. The Transwell^®^ inserts were then washed four times with ddH2O. The non-invasive cells from the upper side of the membrane were removed by carefully sweeping with a cotton swab, and when necessary, excess dye was further removed from the inside and outside of the Transwell^®^ inserts with cotton swabs immersed in ddH2O. The dye was eluted by dipping the Transwell^®^ inserts in 500 µL of a 1% SDS solution. The 1% SDS solution containing the eluted dye was then transferred into a 96-well plate and the absorbance was measured at 650 nm. There were eight replicates of each experiment.

### 2.9. IncuCyte^®^ Migration and Invasion Assays

First, 50 µL of 0.3 mg/mL Myogel were added to the wells of an ImageLock 96-well plate (Essen BioScience, Ann Arbor, MI, USA) and incubated overnight at 37 °C in a 5% CO_2_ humidified atmosphere. The next day, Myogel was aspirated and 25,000 HSC-3 cells in 100 µL of complete media were seeded to each well and incubated overnight. Cells were near 100% confluence the next day. The cell monolayer in the migration assay wells was wounded using the WoundMaker™ 96-pin wound-making tool (Essen BioScience) according to the manufacturer’s instructions. Wounds were inspected under a microscope and fresh culture media was changed. Then, 100 μL of appropriate culture media containing treatments were added to the migration assay wells. Culture media was removed from the invasion assay wells and 50 μL of 2.4 mg/mL Myogel (containing treatments) with 0.2% SeaPlaque™ Low Melting Agarose (LMA) (Lonza, Basel, Switzerland) diluted in PBS were added per well. Any bubbles in the wells were removed using ethanol vapor. The plate was incubated at room temperature for 30 min to let the Myogel-LMA mixture solidify. Then, 50 μL of appropriate media containing treatments or PBS as a control were then added to invasion wells and the plate was placed into IncuCyte ZOOM™. Scans were scheduled to repeat every 2 h for 12 h in the ZOOM software with the scan type set to Scratch wound and Wide mode with the 10x objective. Images were analyzed using IncuCyte ZOOM^®^ 96-Well Scratch Wound Cell Migration and Invasion Data Processing Software Module (Essen BioScience). There were six replicates of each experiment.

### 2.10. Tumor Area and Metastasis in Zebrafish Xenograft

The effect of the bilberry treatment on tumor area and metastasis was investigated in vivo using a zebrafish xenograft. Experiments were conducted in the Zebrafish Unit, University of Helsinki, in compliance with the ARRIVE guidelines (https://arriveguidelines.org; accessed on 5 July 2021). Ethical permission (ESAVI/13139/04.10.05/2017) was given by the regional state administrative agency. Human tongue cancer HSC-3 cells labelled with CellTrace Far red dye (Thermo Fisher Scientific) were microinjected into the perivitelline area of 2-day post fertilization wild-type zebrafish larvae from the AB strain, dechorionated, and anesthetized with 0.04% Tricaine. Each treatment group consisted originally of 20 (experiments 1 and 2) or 25 (experiment 3) zebrafish larvae, each injected with 4 nL (500 cells) of HSC-3 cell suspension.

After injection, zebrafish were transferred to grow on a 24-well plate with 5 fish in 1 mL of fresh embryonic medium per well and treated for 72 h with 2.5, 10, or 25 µg/mL of bilberry powder in embryonic medium, or with embryonic medium only as a control. After treatment, dead larvae were excluded, and live larvae were fixed with 10% paraformaldehyde and mounted in Slowfade Gold Antifade Reagent (Thermo Fisher Scientific) on glass plates for imaging. The experiment was repeated 3 times.

Images were taken with Nikon Eclipse Ti-E Camera (Nikon, Tokyo, Japan) with 10× magnification, and the tumor area in each larva was analyzed with Fiji 2.1.1 (Wayne Rasband, National Institute of Health, Bethesda, MD, USA) [46]. Contrast and brightness were adjusted when needed, similarly between groups, to distinguish tumors accurately. Images with zero signal from labelled tumor cells, potentially due to injection error, were excluded. The number of fish included in the final analysis was 33–38 per group in all 3 repeated experiments combined. Metastasis was analyzed by counting the percentage of fish with tumors outside the perivitelline area (head/tail) in each treatment group.

### 2.11. Statistics

*In vitro* studies: At first, the distribution and normality of the data was tested. Two group means were statistically assessed by Student’s *t*-test. Repeated measures ANOVA was used to compare the mean values between more than two groups with repeated measures, followed by Fisher’s least significant difference (LSD) post hoc test with a Bonferroni correction. Data are presented as mean ± SD and *p* < 0.05 is considered as statistically significant. The data were analyzed using IBM statistic SPSS, version 23.

Tumor area and metastasis in zebrafish xenograft: Differences between treatment group averages from 3 independent experiments were analyzed with one-way Anova with a Bonferroni correction. Data are presented as the mean group averages from 3 independent experiments ± SD, and *p* < 0.05 is considered as statistically significant. The data were analyzed using IBM statistic SPSS, version 27.

## 3. Results

### 3.1. Anthocyanin Composition of the Whole Bilberry Powder

Individual anthocyanins were isolated from whole bilberry powder. Altogether, 24 anthocyanins were recognized (Supplementary Materials Table S1). The highest concentrations were found for Cyanidin 3-*O*-glucoside (226.01 ± 2.74 mg/100 g), Petunidin 3-*O*-glucoside (192.23 ± 4.52 mg/100 g), Peonidin 3-*O*-glucoside (149.69 ± 3.84 mg/100 g), and Delphinidin 3-*O*-glucoside (134.14 ± 3.10 mg/100 g).

### 3.2. Cell Viability

**Figure 1:**
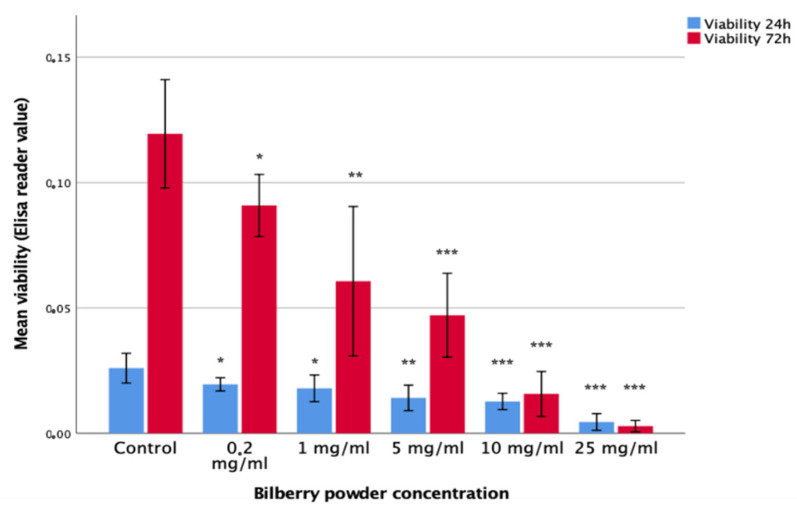
Viability of HSC-3 cells after 24- and 72-h bilberry powder incubations. Statistical test between control and different bilberry powder concentrations: * *p* < 0.05; ** *p* < 0.01; *** *p* < 0.001; 95%CI error bars.

The effects of 0.2, 1, 10, and 25 mg/mL of bilberry powder on the viability of HSC-3 oral carcinoma cells and benign IHGKs and HMKs were studied in 24- and 72-h assays (*n* = 12–18). Bilberry powder at all the studied concentrations of 0.2, 1, 5, 10, and 25 mg/mL decreased the viability of HSC-3 cells significantly (*p* < 0.05) during both assays (24 and 72 h) compared to controls without bilberry powder (Figure 1). The effect of bilberry powder on the viability of benign cell lines was less clear. At the 24-h time point, bilberry powder had no effects on the viability of IHGKs or HMK cells. At the 72-h time point, the two highest concentrations of bilberry powder (10 and 25 mg/mL) decreased viability significantly (*p* < 0.001; Supplementary Materials Table S2).

### 3.3. Cell Proliferation

The effects of 0.2, 1, 10, and 25 mg/mL of bilberry powder on the proliferation of HSC-3 oral carcinoma cells and benign IHGKs and HMKs were studied in 24- and 72-h assays. The highest concentration (25 mg/mL) of bilberry powder inhibited the proliferation of all cell lines significantly (*p* < 0.001) at 24 and 72 h. Bilberry powder at the concentration of 10 mg/mL was capable of inhibiting the proliferation of HSC-3 cells at both time points, whereas the inhibitory effect of bilberry powder on benign cell lines became statistically significant only after the 72-h incubation. The lower concentrations (≤1 mg/mL) of bilberry powder did not inhibit proliferation (Supplementary Materials Table S3).

### 3.4. Transwell Migration

**Figure 2:**
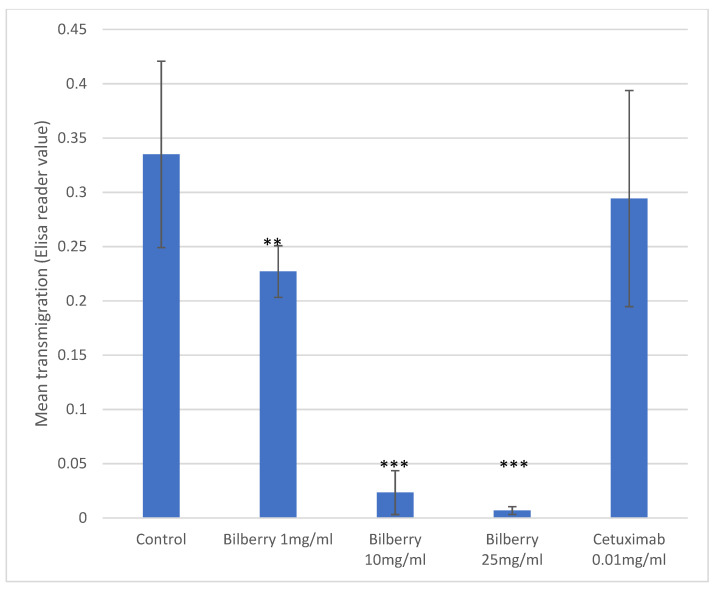
Transmigration of HSC-3 cells. Statistical test between control and different bilberry concentrations and cetuximab: ** *p* < 0.01; *** *p* < 0.001; 95%CI error bars.

In the Transwell assays, HSC-3 cells (*n* = 8) were cultured with 1, 10, and 25 mg/mL of bilberry powder or with 0.01 mg/mL of cetuximab. Bilberry powder decreased the transmigration of the cells significantly with all used concentrations (*p* < 0.01), whereas cetuximab did not (Figure 2).

### 3.5. Incucyte Migration

**Figure 3:**
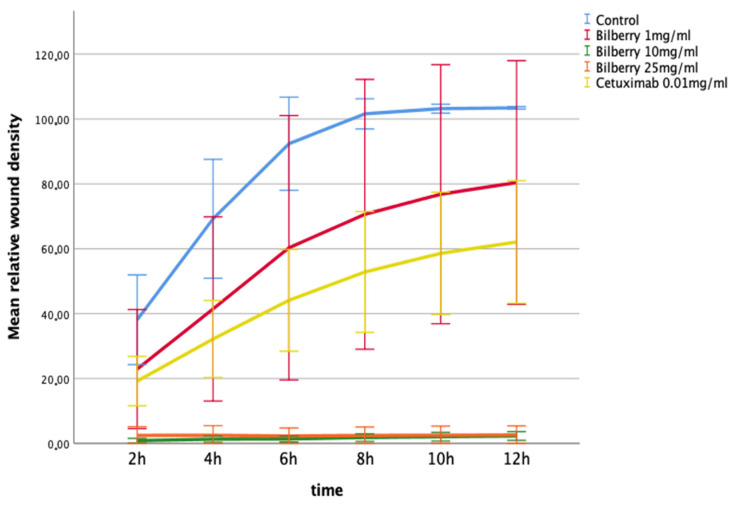
Migration of HSC-3 cells in Myogel after 12 h of bilberry powder and cetuximab incubations. Mean relative wound density and 95%CI error bars.

The effects of 1, 10, and 25 mg/mL of bilberry powder on cell migration were further studied using a real-time cell imaging system (IncuCyte ZOOM^®^ Live-Cell Imaging System, Essen BioScience Inc., Ann Arbor, MICH, USA). In the multivariate test, time and bilberry powder showed significant effects on the migration of the HSC-3 cells (*p* = 0.006). In the least significant difference (LSD) post-hoc test, the two highest concentrations of bilberry powder (10 and 25 mg/mL) differed significantly from the controls (*p* < 0.001). In this assay, cetuximab was also observed to inhibit migration, but statistical significance was lost after Bonferroni correction (*p* > 0.05) (Figure 3).

### 3.6. Incucyte Invasion

**Figure 4:**
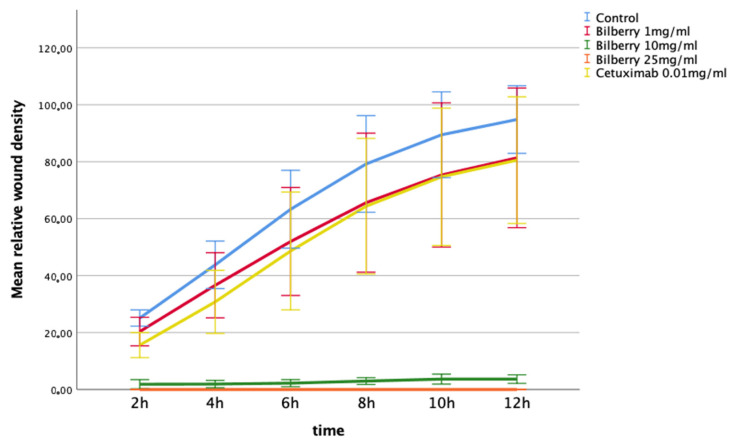
Invasion of HSC-3 cells in Myogel after 12 h of bilberry powder and cetuximab incubations. Mean relative wound density and 95%CI error bars.

The effects of 1, 10, and 25 mg/mL of bilberry powder on cell invasion were also determined using the IncuCyte ZOOM real-time cell imaging system. In the multivariate test, bilberry powder significantly inhibited invasion of the HSC-3 cells (*p* = 0.001). In the LSD post-hoc test, bilberry powder at concentrations of 10 (*p* < 0.001) and 25 mg/mL (*p* < 0.001) inhibited invasion significantly, whereas 0.01 mg/mL of bilberry powder and cetuximab did not (Figure 4).

### 3.7. Tumor Area and Metastasis in Zebrafish Xenograft

**Figure 5:**
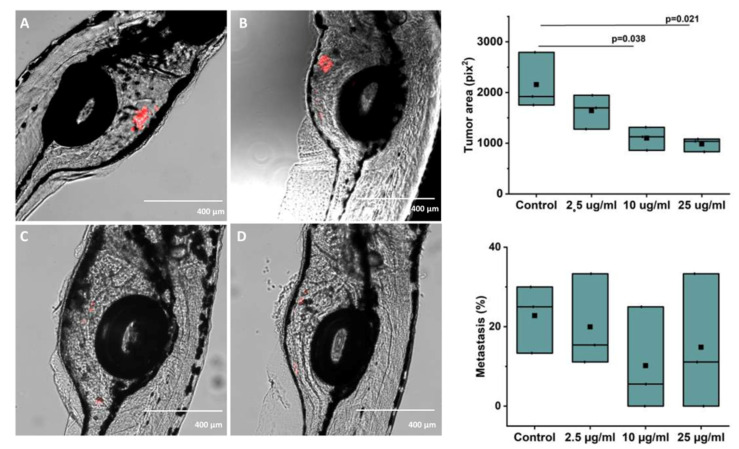
(**A**–**D**): Representative images of zebrafish microinjected with labelled HSC-3 cells and treated with (**A**): Control (embryonic media only), (**B**) 2.5 μg/mL, (**C**) 10 μg/mL, or (**D**) 25 μg/mL of whole bilberry powder in embryonic media. E: Tumor area and F: Metastasis as group averages from 3 independent experiments (33–38 fish per group in the 3 experiments combined) after treatment with different concentrations of bilberry powder.

Bilberry treatment significantly reduced the tumor area compared to the control with 10 and 25 µg/mL of bilberry powder (*p* = 0.038 and *p* = 0.021, respectively, Figure 5). The group averages of tumor area from 3 independent experiments were 2155.77 ± 322.06 pix^2^ for controls, 1640.45 ± 194.96 pix^2^ for fish treated with 2.5 µg/mL, 1100.67 ± 132.21 pix^2^ for fish treated with 10 µg/mL, and 983.80 ± 77.46 pix^2^ for fish treated with 25 µg/mL of bilberry powder. Metastasis was not significantly affected; the group average for the percentage of fish with metastasis was 22.78 ± 4.94% in controls, 19.94 ± 6.80% in fish treated with 2.5 µg/mL, 10.18 ± 7.58% in fish treated with 10 µg/mL, and 14.81 ± 9.80% in fish treated with 25 µg/mL of bilberry powder (Figure 5).

## 4. Discussion

This is the first study to demonstrate that whole bilberry powder exhibits anticarcinogenic activity on human oral squamous carcinoma cells in vitro and in vivo. Bilberry powder inhibited the fundamental behaviors of oral cancer cells, including viability, proliferation, migration, and invasion. These inhibitory effects were more pronounced with higher bilberry powder concentrations as well as in malignant cells compared with benign cells. Moreover, compared with cetuximab, bilberry powder was observed to have comparable effects, and even more potent effects in a dose-dependent manner. The in vivo tumor-inhibiting activity of bilberry powder was confirmed in the zebrafish xenograft; however, statistically significant inhibition of metastasis was not reached in this experiment.

The genus *Vaccinium* includes both wild and cultivated species, e.g., bilberry, blueberry, and cranberry, which are all well known for their health-promoting effects [24]. In comparison with other *Vaccinium* species, the anthocyanin content is especially high in wild-growing bilberry, and it has been shown to be one of the richest natural sources of anthocyanins [23,24,25,26,28,29]. Anthocyanins are derived from anthocyanidins with additional sugar and/or acyl moieties in different positions, and, together with other phenolic compounds in bilberry, are linked to many biological activities, including anticancer effects [24,29,30]. Based on their anthocyanin content, antioxidant-rich bilberry could be more effective than cultivated *Vaccinium* species also in the context of pre-/malignant oral epithelial lesions. However, the anticancer activity of bilberry is likely shared with phytochemicals other than anthocyanins [16] and this warrants further studies.

Using whole bilberry powder in medical applications would require tracing the origin, content, and authenticity of the berries, particularly as the concentration of anthocyanins varies by geographic regions [31,32]. In most studies, the anthocyanin content of whole bilberry extract has not been analyzed and the collection area or origin not reported. This makes it difficult to compare the anthocyanidin content in our powder with bilberry extracts used in other in vitro or in vivo cancer studies.

In this study, the anthocyanin content in whole bilberry powder was analyzed, and data are provided in detail in Supplementary Materials Table S1. The highest concentrations were found for the 3-*O*-glucosides of Cyanidin, Paeonidin, Delphinidin, and Petunidin, respectively. Previous studies of the anthocyanin profiles in bilberries have reported the same derivatives but in different concentrations [43]. In native bilberry populations in the Alps of northern Italy, Delphinidin 3-*O*-glucoside was found to be markedly higher while the other three major compounds were lower in their concentrations [47]. Similar variation of the anthocyanin pattern was reported in bilberry fruits collected all over Europe, including Finland [48].

In the present study, bilberry powder inhibited vertical migration of HSC-3 cells in the Transwell assay as well as horizontal migration across the Myogel in the Incucyte migration assay, which was statistically significant and in a dose-dependent manner. The results are in line with previous studies demonstrating blueberry juice and powder to inhibit migration of various human carcinoma cell lines as well as hypoxia-induced migration of endothelial cells [12,15]. Moreover, blueberry suppressed invasion [12]. In the in vivo assay of our study, the in vitro results were confirmed as the area of HSC-3 tumors was significantly reduced in zebrafish growing in embryonic medium with bilberry powder compared to controls. These results are in accordance with a previous in vivo study, in which a shift to an anti-invasive oral carcinoma phenotype with less hyperplasia and dysplasia was induced in bilberry-supplemented hamsters [17]. On the other hand, we did not achieve significant inhibition of metastasis in our zebrafish xenograft experiment. This could be due to the low metastasis rate observed in this three-day experiment, or to the differential behavior of cancer cells injected into the perivitelline area of zebrafish compared to cancer cells located in the oral mucosa of mammals.

Bilberry powder was also compared with cetuximab in the migration and invasion assays. Cetuximab (Erbitux^®^) is a chimeric monoclonal antibody of epidermal growth factor receptor (EGFR) [49]. EGFR is expressed in normal oral epithelium as well as in most oral squamous carcinoma cell lines [33,50]. By competitively blocking the EGFR, cetuximab can affect the cell cycle and promote apoptosis [33]. In the current study, cetuximab was observed to inhibit migration significantly (*p* = 0.02) in the IncuCyte migration assay. Somewhat unexpectedly, and in contrast to bilberry powder, cetuximab had no effect on invasion in the Incucyte assay or migration in the Transwell assay. Nonetheless, previous in vitro studies as well as clinical observations have suggested that EGFR signaling may affect cell migration in a cell type-dependent manner and not all cancer cells express EGFR [50].

The antiproliferative activity of bilberry on oral cancer cells has not been previously investigated, but a body of evidence exists on the growth-inhibiting effects of bilberry on colorectal cancer [16]. Similarly, blueberry inhibits the proliferation of non-oral carcinoma cells and carcinoma stem cells [14,15,51]. Cranberry, another berry species of the genus *Vaccinium*, has been shown to have antiproliferative effects on CAL27 and SCC25 oral squamous carcinoma cells [52]. Fermented lingonberry juice also inhibited proliferation of SCC25, in addition to HSC-3 [18], which were also used in our experiments. Similar results on the effects of anthocyanin-rich black raspberry on oral cancer cell proliferation have been obtained [53,54]. In addition, treatment of oral mucosal dysplastic lesions of human patients topically with a 10% black raspberry gel, four times per day for 6 weeks, induced histologic regression of about 60% of the dysplastic lesions [22]. Additionally, in the present study, bilberry powder inhibited the proliferation of both malignant cells and benign but dysplastic oral epithelial cells. However, bilberry powder inhibited the proliferation of HSC-3 tongue carcinoma cells with a lower concentration than that of benign cell lines, suggesting stronger inhibition of malignant cells.

Inhibition of tumor growth was confirmed in the zebrafish xenograft, and inhibition without noticeable toxicity also supports a stronger effect of bilberry on tumorigenic than normal cells. Results from this non-mammal model should not be generalized without caution, but they are in line with previous experiments with bilberry on various murine models of colorectal cancer [55,56,57,58,59,60,61]. One limitation of our zebrafish method was analyzing the tumor area from 2-D images, which does not accurately depict the volume of the tumor. However, the tumor area was reduced in a dose-dependent manner by bilberry treatment, and the difference from the control was significant with the 10 and 25 µg/mL concentrations. Measuring the tumor volume instead of area would likely have shown even larger differences between the study groups.

The effective concentrations of bilberry powder used in the present study are relatively high to be consumed for therapeutic responses in humans. However, tissues in the digestive tract can be exposed to higher quantities of ingested compounds than tissues that rely only on systemic delivery, and accumulation of phenolic compounds into tissues over time has been reported [55,62]. In this study, effective concentrations were lower in vivo than in cell culture (micrograms versus milligrams per milliliter), possibly indicating a stronger effect on cancer cells in a living organism compared to cell culture. Additionally, bilberry toxicity is not known to be an issue in humans, and topical preparations similar to black raspberry gel could be developed [22].

Nonetheless, studies have been addressed to develop berry extracts for efficient systemic delivery and better bioavailability. Recent studies have particularly focused on anthocyanins because of their well-documented anticancer effects in in vitro and in vivo models [63]. However, anthocyanins are hydrophilic compounds, and thus unable to cross the cell membrane by passive diffusion. Different approaches to enhance the bioavailability of anthocyanins have been taken, and one study showed that bilberry extract does have an increased effect on human erythroleukemic cancer cells when it is encapsulated in NutraNanoSpheres, compared to the free compound [64]. In another recent study, nano-formulations of bilberry-derived anthocyanidins packed in exosomes were used [65]. Exosomal formulation of anthocyanidins enhanced the antiproliferative and anti-inflammatory effects compared with the free metabolite against lung, breast, colon, pancreatic, prostate, and ovarium cancer cells in vitro. These innovative bilberry extracts could also provide more efficient oral delivery of anthocyanins to oral cancer patients. However, a too reduced approach may not bring the best effect. Anthocyanin mixture has been shown to inhibit small-cell lung cancer cell proliferation and metastasis significantly more than its individual compounds [66], and other phenolic compounds, such as procyanidins, inhibit proliferation of colorectal cancer cells [67,68,69,70].

## 5. Conclusions

Whole bilberry powder inhibited the migration, invasion, and proliferation of oral squamous carcinoma in vitro, and tumor growth in vivo, indicating potential for clinical trials in oral cancer prevention and treatment.

## Data Availability

The data presented in this study are available on request from the corresponding author. The data are not publicly available due to ethical reasons of data storage.

## Supplementary Materials

The following are available online at https://www.mdpi.com/article/10.3390/antiox10081319/s1. Table S1. Average amount of AC content (±SD; mg/100 g; dry weight) of the bilberry powder used at the assays. Dp, Delphinidin; Cy, Cyanidin; Pt, Petunidin; Pn, Peonidin; Pl, Pelargonidin; Mv, Malvidin. Table S2. The effects of wild blueberry powder (0, 0,2, 1, 5, 10 and 25 mg/mL) on the viability of HSC-3, IHGKs and HMKs after 24 h and 72 h incubations. Table S3. The effects of wild blueberry powder (0, 0,2, 1, 5, 10 and 25 mg/mL) on the proliferation of HSC-3, IHGKs and HMKs after 24 h and 72 h incubations.
