# Supplementary material for: Bilberry (Vaccinium myrtillus L.) Powder Has Anticarcinogenic Effects on Oral Carcinoma In Vitro and In Vivo
Source: Antioxidants (Basel). 2021 Aug 22;10(8):1319. doi: 10.3390/antiox10081319 (PMC8389301; doi:10.3390/antiox10081319)
Supplement: Supplementary file 1 [file antioxidants-10-01319-s001.zip › antioxidants-1309116-supplementary.pdf]

Supplementary Table S1. Average amount of AC content (± SD; mg/100 g; dry weight) of the bilberry powder used at the assays. Dp, Delphinidin; Cy, Cyanidin; Pt, Petunidin; Pn, Peonidin; Pl, Pelargonidin; Mv, Malvidin.

Compounds	mg/100g
**Dp 3-glc**	134.14 ± 3.10
**Cy 3 glc**	226.01± 2.74
**Pt 3-glc**	192.23± 4.52
**Pn 3-glc**	149.69± 3.84
**Pl 3-glc**	2.44± 0.28
**Mv 3-glc**	132.17± 5.11
**Pn p-coumaryl 3-glu**	0.57± 0.09
**Mv p-coumaryl 3-glu**	0.00
**Dp-3-arab**	97.26± 3.17
**Cy 3-arab**	109.48± 2.31
**Pt-3-arab**	37.72± 1.13
**Pn 3-arab**	18.43± 0.43
**Mv-3-arab**	39.21± 1.12
**Cy-3-rut**	6.96± 3.73
**Dp 3-gal**	56.01± 0.9
**Cy 3-gal**	31.56± 15.83
**Pt 3-gal**	60.37± 1.95
**Pn 3-gal**	78.28± 3.07
**Mv 3-gal**	62.26± 1.61
**Pl 3-gal**	0.81± 0.05
**Cy 3-samb**	9.76 ± 0.39
**Cy-3,5-diglucoside**	0.81 ± 0.05
**Pn-3,5-diglucoside**	0.4 ± 0.06

Supplementary Table S2. The effects of wild blueberry powder (0, 0,2, 1, 5, 10 and 25 mg/ml) on the viability of HSC-3, IHGKs and HMKs after 24h and 72h incubations.

		N	Mean	Std. Dev.	T-test (Sig)
**HSC-3 24h**	Control	12	0,03	0,01	
	0,2 mg/ml	12	0,02	0,00	**0,04**
	1 mg/ml	12	0,02	0,01	**0,04**
	5 mg/ml	12	0,01	0,01	**0,003**
	10 mg/ml	12	0,01	0,01	**0,000**
	25 mg/ml	12	0,00	0,01	**0,000**
**HSC-3 72h**	Control	12	0,12	0,03	
	0,2 mg/ml	12	0,09	0,02	**0,02**
	1 mg/ml	12	0,06	0,05	**0,002**
	5 mg/ml	12	0,05	0,03	**0,000**
	10 mg/ml	12	0,02	0,01	**0,000**
	25 mg/ml	12	0,00	0,00	**0,000**
**IHGK 24h**	Control	18	0,02	0,02	
	0,2 mg/ml	18	0,02	0,02	0,640
	1 mg/ml	18	0,03	0,02	0,406
	5 mg/ml	17	0,03	0,02	0,186
	10 mg/ml	17	0,04	0,03	0,065
	25 mg/ml	17	0,02	0,01	0,885
**IHGK 72h**	Control	18	0,05	0,02	
	0,2 mg/ml	18	0,07	0,02	**0,005**
	1 mg/ml	18	0,07	0,02	**0,003**
	5 mg/ml	17	0,05	0,02	0,558

	10 mg/ml	17	0,02	0,01	**0,000**
	25 mg/ml	17	0,02	0,01	**0,000**
**HMK 24h**	Control	12	0,01	0,01	
	0,2 mg/ml	12	0,01	0,01	0,820
	1 mg/ml	12	0,01	0,01	0,707
	5 mg/ml	12	0,01	0,01	0,442
	10 mg/ml	12	0,01	0,01	0,280
	25 mg/ml	12	0,01	0,01	0,104
**HMK 72h**	Control	18	0,10	0,04	
	0,2 mg/ml	18	0,10	0,05	0,960
	1 mg/ml	18	0,09	0,04	0,712
	5 mg/ml	18	0,10	0,04	0,771
	10 mg/ml	18	0,05	0,03	**0,000**
	25 mg/ml	18	0,02	0,01	**0,000**

Supplementary Table S3. The effects of wild blueberry powder (0, 0,2, 1, 5, 10 and 25 mg/ml) on the proliferation of HSC-3, IHGKs and HMKs after 24h and 72h incubations.

		N	Mean	Std. Dev.	T-test (Sig)
**HSC-3 24h**	Control	12	0,80	0,08	
	0,2 mg/ml	12	1,08	0,26	**0,01**
	1 mg/ml	11	0,90	0,27	0,35
	5 mg/ml	12	0,72	0,29	0,49
	10 mg/ml	12	0,41	0,24	**0,00**
	25 mg/ml	8	0,00	0,00	**0,00**
**HSC-3 72h**	Control	12	0,57	0,28	
	0,2 mg/ml	11	0,61	0,36	0,76
	1 mg/ml	11	0,84	0,66	0,29
	5 mg/ml	12	0,80	0,53	0,27
	10 mg/ml	12	0,24	0,23	**0,01**
	25 mg/ml	12	0,00	0,01	**0,00**
**IHGK 24h**	Control	18	0,16	0,07	
	0,2 mg/ml	18	0,18	0,08	0,544
	1 mg/ml	18	0,17	0,07	0,874
	5 mg/ml	18	0,16	0,08	0,923
	10 mg/ml	18	0,14	0,05	0,220
	25 mg/ml	18	0,05	0,05	**0,000**
**IHKG 72h**	Control	18	0,87	0,56	
	0,2 mg/ml	18	0,96	0,68	0,687
	1 mg/ml	18	0,88	0,58	0,951
	5 mg/ml	18	0,56	0,46	0,075
	10 mg/ml	18	0,28	0,25	**0,000**

	25 mg/ml	18	0,11	0,04	**0,000**
**HMK 24h**	Control	18	0,33	0,11	
	0,2 mg/ml	18	0,29	0,07	0,216
	1 mg/ml	18	0,34	0,16	0,865
	5 mg/ml	18	0,32	0,10	0,803
	10 mg/ml	18	0,29	0,11	0,339
	25 mg/ml	18	0,04	0,01	**0,000**
**HMK 72h**	Control	18	0,57	0,12	
	0,2 mg/ml	18	0,55	0,14	0,621
	1 mg/ml	18	0,65	0,24	0,212
	5 mg/ml	18	0,49	0,40	0,418
	10 mg/ml	18	0,13	0,12	**0,000**
	25 mg/ml	18	0,11	0,03	**0,000**
